# The Authentication of Java Turmeric (*Curcuma xanthorrhiza*) Using Thin Layer Chromatography and ^1^H-NMR Based-Metabolite Fingerprinting Coupled with Multivariate Analysis

**DOI:** 10.3390/molecules25173928

**Published:** 2020-08-27

**Authors:** Abdul Rohman, Theresia Wijayanti, Anjar Windarsih, Sugeng Riyanto

**Affiliations:** 1 Department of Pharmaceutical Chemistry, Faculty of Pharmacy, Universitas Gadjah Mada, Yogyakarta 55281, Indonesia; terewijaya@gmail.com (T.W.); sugeng_riyanto@ugm.ac.id (S.R.); 2 Institute of Halal Industry and Systems (IHIS), Universitas Gadjah Mada, Yogyakarta 55281, Indonesia; 3 Research Division for Natural Product Technology (BPTBA), Indonesian Institute of Sciences (LIPI), Yogyakarta 55861, Indonesia; anjarwindarsih2@gmail.com

**Keywords:** authentication, *Curcuma xanthorrhiza*, ^1^H-NMR, OPLS-DA, PCA, TLC

## Abstract

The identification of adulteration practices of medicinal plants used as herbal medicine is very important to ensure the quality, safety, and efficacy. In this study, thin layer chromatography (TLC) and proton nuclear magnetic resonance (^1^H-NMR)-based metabolite fingerprinting coupled with multivariate analysis were used for authentication of *Curcuma xanthorrhiza* extract from *Curcuma aeruginosa*. Curcumin contents obtained from *C. xanthorrhiza* extract from various regions were in the range of 0.74%–1.23%. Meanwhile, curcumin contents obtained from *C. xanthorrhiza* extract adulterated with 0%, 10%, 25%, 40%, 50%, and 75% of *C. aeruginosa* were 1.02%, 0.96%, 0.86%, 0.69%, 0.43%, and 0.27%, respectively. The decreasing of curcumin contents in adulterant concentrations of 40% and more in *C. xanthorrhiza* rhizome could indicate the adulteration with other rhizomes. Multivariate analysis of PCA (principal component analysis) using data set obtained from ^1^H-NMR spectra clearly discriminated pure and adulterated *C. xanthorrhiza* with *C. aeruginosa*. OPLS-DA (orthogonal projections to latent structures-discriminant analysis) successfully classified pure and adulterated *C. xanthorrhiza* with higher R2X (0.965), R2Y (0.958), and Q2(cum) (0.93). It can be concluded that ^1^H-NMR-based metabolite fingerprinting coupled with PCA and OPLS-DA offers an adequate method to assess adulteration practice and to evaluate the authentication of *C. xanthorrhiza* extracts.

## 1. Introduction

*Curcuma xanthorrhiza*, also known as Javanese turmeric or *Temulawak,* belongs to family Zingiberaceae and is a native medicinal plant from Indonesia [1]. This plant is widely cultivated around the world, especially in Southeast Asia, due to its variety of pharmacological activities, such as anticancer, antimicrobial, anti-inflammatory, antioxidant, anti-candida, anti-hyperglycemic, and antihypertensive effects [2,3]. Most of potential pharmacological activities of *C. xanthorrhiza* are believed to come from various bioactive and phytochemical compounds, including xanthorrhizol (1.48%–1.63%); curcuminoids, such as curcumin and demethoxycurcumin (1%–2%); phelandren; camphor; tumerol; sineol; borneol; flavonoids; and sesquiterpenes [4]. Traditionally, this herb is utilized as home remedies, such as *jamu prescriptions*, food supplements, and herbal drinks, but recently, this plant has also been gaining popularity and is extensively used as raw material in pharmaceutical and cosmetic industries [5]. 

Due to high demand for *C. xanthorrhiza* in the herbal industries and the price of this herb normally being higher than other herbs in the traditional market in Indonesia, adulteration commonly occurs, and this has caused a major problem [6,7]. The practice of adulteration, either intentionally or unintentionally, usually includes partial or full substitution of an original crude mixture of medicinal plants with other substances which are cheaper, and either free from or inferior in therapeutic properties [8]. The most common rhizome used as a *C. xanthorrhiza* adulterant is *C. aeruginosa* (which also belongs to family Zingiberaceae) due to the similar colors and similar biological activities of the two rhizomes [7].

The authentication of *C. xanthorrhiza* is necessary to be carried out in order to ensure the quality, safety, and efficacy of raw material and its finished products [9]. Several analytical methods have been reported which applied the fingerprinting analytical approach using high performance liquid chromatography with a diode array detector [9], gas chromatography-time of flight mass spectrometry, and ultrahigh-performance liquid chromatography-quadrupole time-of-flight mass spectrometry [10]; capillary electrophoresis [11]; and high performance thin layer chromatography [12]. Thin layer chromatography (TLC) method has been developed for analysis of drugs and medicinal plants including *Curcuma* species. TLC could be used for qualitative and semiquantitative analysis, required less organic solvent, and required less time for analysis. Moreover, optimization of the TLC system could be performed in curcumin analysis for medicinal plant authentication [13]. The latter metabolomic approach using ^1^H-NMR-based metabolite fingerprinting is gaining popularity in the field of discrimination and authentication of medicinal plants. This method offers simultaneous identifications of various groups of secondary and primary metabolites [14,15]. In addition, this method is powerful, fast, reproducible, requires minimal sample preparation, and could be used to analyze metabolite profiles with only a crude extract [16,17]. Combined with chemometrics such as principal component analysis (PCA), partial least square-discriminant analysis (PLS-DA), and orthogonal projections to latent structures-discriminant analysis (OPLS-DA), the huge datasets generated from ^1^H-NMR measurements could be handled [18,19] and can be used to assess the authentication of *C. xanthorrhiza* extract.

In the best of our knowledge, there is no publication regarding the authentication of *C. xanthorrhiza* extract adulterated with *C. aeruginosa*. In this study, a thin layer chromatography (TLC) method was validated and employed for the determination of curcumin contents in *C. xanthorrhiza* extracts. The curcumin contents in *C. xanthorrhiza* could be used to evaluate the adulteration indication of this extract. The objective of this study was to develop thin layer chromatography (TLC) and proton nuclear magnetic resonance (^1^H-NMR)-based metabolite fingerprinting coupled with multivariate analysis of PCA and OPLS-DA for the authentication and discrimination of *C. xanthorrhiza* extract from *C. aeruginosa*.

## 2. Results and Discussion

### 2.1. Analytical Method Validation

**Figure 1:**
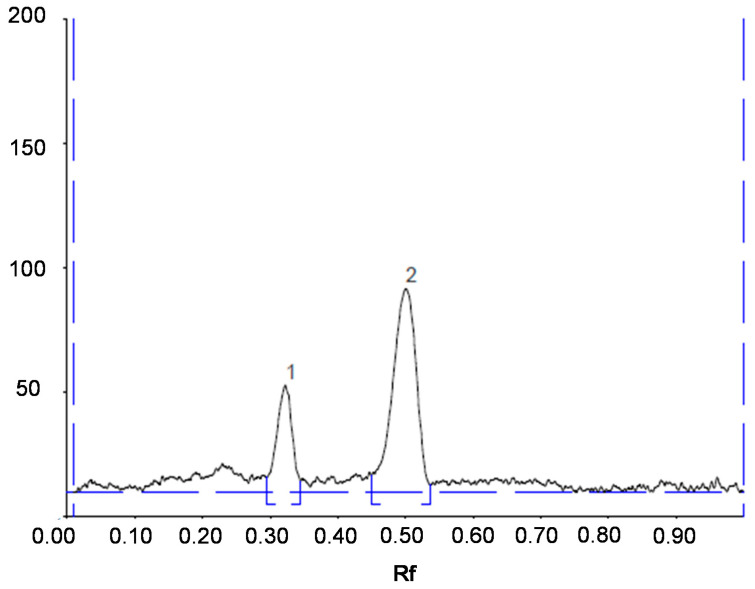
Chromatogram of *Curcuma xanthorrhiza* sample. Chromatographic conditions: mobile phase, chloroform:methanol:formic acid (94:3:3 *v*/*v*); (1: demethoxycurcumin; 2: curcumin).

Validation of TLC method was performed by determining the performance characteristics which included selectivity, linearity and range, detection limit and quantitation limit, accuracy, and precision according to the International Conference on Harmonization (ICH) Q2(R1). Selectivity test was conducted on *C. xanthorrhiza* sample from Bantul. Resolution (Rs) value was calculated to assess the separation of curcumin peak from other peaks. The retention factor (Rf) value of curcumin was 0.50; meanwhile, the Rf value of other peak suspected as demethoxycurcumin was 0.32, and the mean Rs value of curcumin obtained was 2.43, indicating that curcumin peak was fully separated from other peaks, as can be seen in Figure 1. Thus, it can be concluded that the developed method is selective for curcumin determination. 

In a linearity test, the peak area versus concentration of curcumin standard was plotted to obtain the calibration curve equation and correlation coefficient. The calibration curve equation of the curcumin obtained was y = 45.883x + 6042.9 with a correlation coefficient (r) of 0.9972. This method is linear in the range of 250–450 ng. Detection limit and quantitation limit of curcumin were 20.62 ng and 68.72 ng respectively. These results were obtained based on the standard deviation approach of the calibration curve. Accuracy and precision were assessed in the sample by standard addition method. These tests were performed at three concentration levels (low, medium, high) with three replications of each concentration. The accuracy and precision of this method were determined by calculating the recovery and relative standard deviation (RSD) value, respectively. Table 1 showed the results of the accuracy and precision study of the sample by the standard addition method. The three concentration levels of addition consisted of low level (10 µg/mL), medium level (12.5 µg/mL), and high level (15 µg/mL). The mean value of recovery obtained was within the required range of 80%–110% for low level and 90%–107% for the medium and high level [20]. The RSD percentages (the precision parameter) at three concentration levels were below the maximum limit of Horwitz’s RSD value which is 11.3% for the low level and 8% for the medium and high levels. These results of this study showed that this method is highly precise and accurate for determining curcumin in all concentration levels.

### 2.2. Determination of Curcumin Contents in the Samples

**Figure 2:**
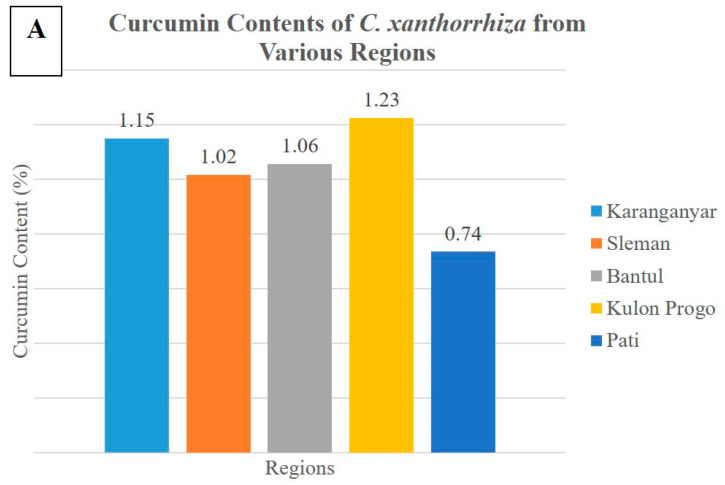

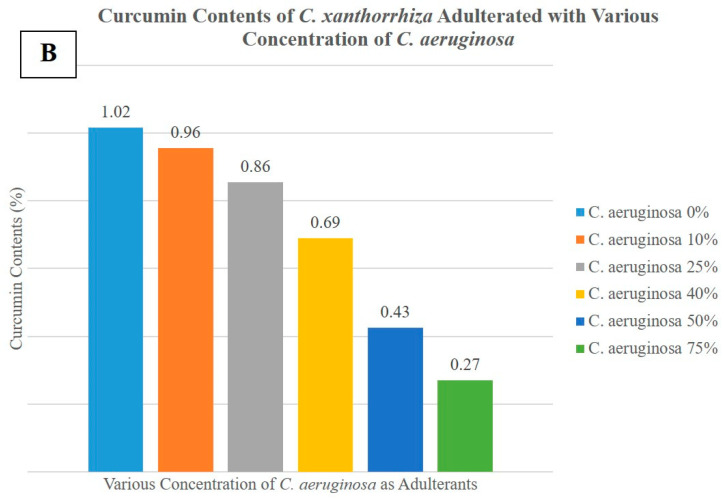
Curcumin contents of (**A**) *C. xanthorrhiza* from various regions; (**B**) *C. xanthorrhiza* adulterated with various concentrations of *C. aeruginosa*.

Table 2 shows the curcumin contents of *C. xanthorrhiza* extract from various regions and the curcumin contents of *C. xanthorrhiza* adulterated with various concentration of *C. aeruginosa*. The percentages of RSD obtained from all samples meet the acceptance criteria according to Horwitz’s RSD, which is below 4% [20]. Curcumin contents obtained from *C. xanthorrhiza* extract from various regions were in the range of 0.74%–1.23%, as can be seen in Figure 2A. Meanwhile, curcumin contents obtained from *C. xanthorrhiza* extract adulterated with 0%, 10%, 25%, 40%, 50%, and 75% of *C. aeruginosa* were 1.02%, 0.96%, 0.86%, 0.69%, 0.43%, and 0.27% respectively (Figure 2B). There is a decrease in the level of curcumin content with an increasing level of *C. aeruginosa* (the adulterant) added. 

The decreasing of curcumin contents in the samples of *C. xanthorrhiza* rhizome could indicate that these samples are adulterated with another rhizome which has a lower concentration of curcumin; in this case, *C. aeruginosa* rhizome. The curcumin concentration in *C. aeruginosa* was around 0.01%–0.57% [21] so that it would decrease the curcumin content when it was used as an adulterant in *C. xanthorrhiza*. Therefore, curcumin concentration could be used as a parameter for quality control of *C. xanthorrhiza* powdered rhizome to ensure its authenticity. The determination of curcumin contents using TLC method could be used to assess *C. xanthorrhiza* adulteration, especially at adulterant concentrations of 40% and higher.

### 2.3. ^1^H-NMR Spectra Analysis

**Figure 3:**
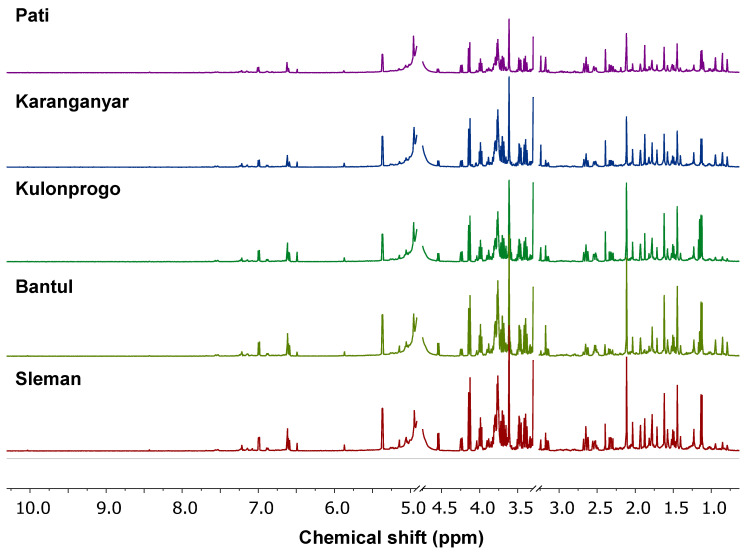
^1^H-NMR spectra of *C. xanthorrhiza* from several regions.

Representative one-dimensional ^1^H-NMR spectra of *C. xanthorrhiza* obtained from several regions are presented in Figure 3. The used of phosphate buffer (KH_2_PO_4_) in D_2_O was to maintain pH value of the compounds because the stable pH value is important to maintaining the stability of the compounds; therefore, it offers better resolution spectra. Moreover, TSP 0.01% was for chemical shift calibration of ^1^H-NMR signals. Generally, the ^1^H-NMR spectra of plant extract could be divided into three categories; namely, the organic acid and amino acid region (chemical shifts of 0.00–3.00 ppm), the carbohydrates/glucose region (chemical shifts of 3.01–5.00 ppm), and the aromatic region (chemical shifts of 6.00–8.00 ppm) [15]. ^1^H-NMR spectra are considered fingerprint spectra because there are no samples with identical ^1^H-NMR spectra. All *C. xanthorrhiza* samples from several regions presented similar ^1^H-NMR spectral patterns in all categorized areas (amino/organic acid, carbohydrate/glucose, and aromatic) as can be seen in Figure 3, even though there are some differences either in signal pattern or in intensities. It indicated that most of the metabolites in *C. xanthorrhiza* from several regions were similar.

**Figure 4:**
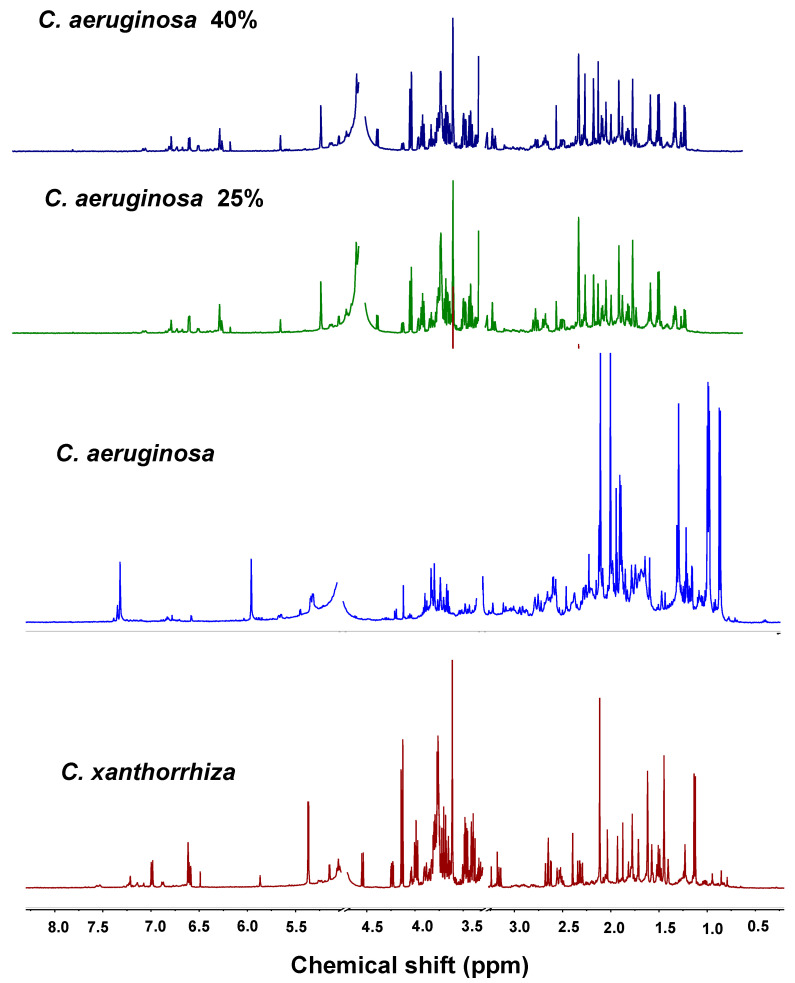
^1^H-NMR spectra of pure *C. xanthorrhiza*, pure *C. aeruginosa*, and adulterated *C. xanthorrhiza* with *C. aeruginosa*.

The ^1^H-NMR spectra of *C. xanthorrhiza* differ from ^1^H-NMR spectra of *C. aeruginosa* in several chemical shift regions (Figure 4). *C. aeruginosa* showed higher contents of organic acid/amino acid compared to *C. xanthorrhiza* indicated by higher intensity of the spectra in the region of 0.00–3.00 ppm. On the other hand, the glucose/carbohydrate content of *C. aeruginosa* is very low compared to that in *C. xanthorrhiza*. In the aromatic region (6.00–8.00 ppm), *C. xanthorrhiza* has higher signal intensity compared to *C. aeruginosa*, especially in the curcumin region which indicated higher curcumin concentration in *C. xanthorrhiza*. Curcumin could be identified at 7.28 ppm (singlet), 3.65 ppm (singlet), 6.77 ppm (doublet), 7.17 ppm (doublet), and 7.56 ppm (singlet), whereas the signals of demethoxycurcumin appeared at 3.66 ppm (singlet), 5.86 ppm (singlet), and 6.92 ppm (doublet).

Even though the ^1^H-NMR spectra of pure *C. xanthorrhiza* differ from the spectra of pure *C. aeruginosa*, in adulterated samples, it was very difficult to state whether the spectra were from authentic or adulterated samples because they possess very similar spectra. The spectra of adulterated *C. xanthorrhiza* with 25% of *C. aeruginosa* and even in 40% concentration of *C. aeruginosa* still showed very similar ^1^H-NMR spectra with authentic spectra of *C. xanthorrhiza* (Figure 4). A deep investigation could distinguish them by such things as decreasing intensities in the aromatic and carbohydrate regions and the spectral changes in the amino/organic acid region. However, it possibly makes the mistake of justifying the samples when there is no information regarding to the samples. Therefore, an adequate statistical method such as multivariate analysis is needed to overcome these problems.

### 2.4. Multivariate Analysis

The ASCII data generated from ^1^H-NMR spectra were Pareto scaled before multivariate analysis. Pareto scaling was used to diminish the effects of variables that do not have important roles in classification. The strong signal intensities which do not have significant influences on classification were removed while the weak signals with significant influence were taken to obtain optimum separation. ^1^H-NMR data were subjected to PCA to visualize the dissimilarities and similarities among samples via a PCA score plot. It can be used to predict adulteration in *C. xanthorrhiza* samples with *C. aeruginosa*. PCA is unsupervised pattern recognition method used to decrease the amount of multivariate data set when there is a correlation [15,22,23]. By employing PCA, all samples were grouped with the maximum separation based on the signal intensities in the ^1^H-NMR spectra representing the metabolites [24].

**Figure 5:**
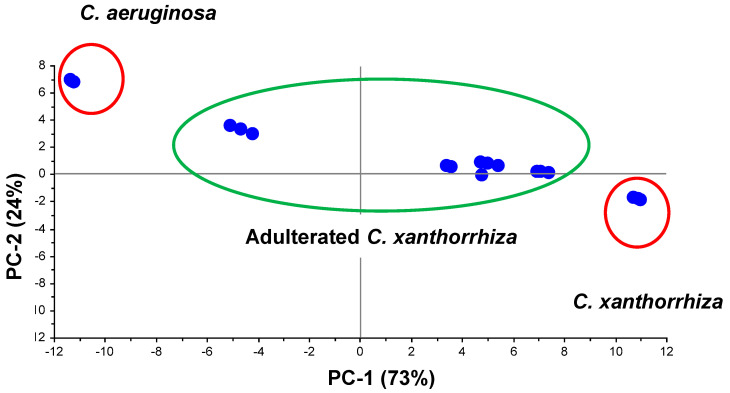
PCA score plot of pure *C. xanthorrhiza*, adulterated *C. xanthorrhiza*, and pure *C. aeruginosa*.

PCA using two principal components (PC) showed a clear separation between pure *C. xanthorrhiza*, pure *C. aeruginosa*, and adulterated *C. xanthorrhiza* using several concentration levels of *C. aeruginosa* (Figure 5). Hundreds of original variables used for making PCA model were reduced to be principal components, which explains the original variables. PC1 and PC2 could represent 97% of variance explained by 73% of PC1 and 24% of PC2. The scores appearing close to each other in PCA indicated more similarities among them. Samples having identical characteristics overlapped in the same score plot. *C. xanthorrhiza* samples with higher adulterant (*C. aeruginosa*) concentration appeared close to pure *C. aeruginosa*, indicating that the higher the adulterant concentration, the higher the similarities to adulterant in metabolite contents.

**Figure 6:**
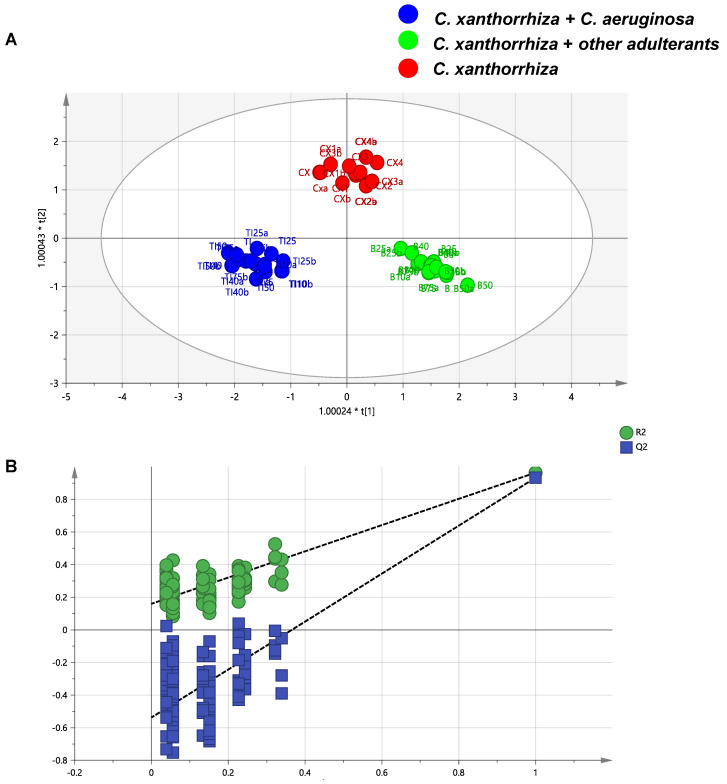
OPLS-DA score plot of pure and adulterated *C. xanthorrhiza* (**A**) and permutation test of OPLS-DA model (**B**).

Another multivariate analysis technique, namely, OPLS-DA, was subjected to differentiate pure *C. xanthorrhiza* and adulterated *C. xanthorrhiza* with *C. aeruginosa* and other species. OPLS-DA is supervised pattern recognition which uses orthogonal X and Y variables for classification. OPLS-DA using two predictive and six orthogonal components revealed complete differentiation between pure and adulterated samples of *C. xanthorrhiza* presented in OPLS-DA score plot (Figure 6A). The OPLS-DA model had higher R2X (0.965), R2Y (0.958), and Q2(cum) (0.93) values. Higher R2X and R2Y values (close to 1) indicated good of fitness of the model. Meanwhile, the strength of the predictivity of the model was evaluated by Q2(cum) value. The higher Q2(cum) value (more than 0.5) indicated the strong predictivity of the developed model [25,26]. Supervised pattern recognition including OPLS-DA needed to be validated. A permutation test was carried out to validate the OPLS-DA model (Figure 6B). The X variables were kept intact while the Y variables were permuted during a permutation test. The R2 and Q2 models of Y variables were permutated 100 times (on the left side) and then compared to the original model (on the right side). To be categorized as a valid model, the original model of R2 and Q2 must be higher than all the permutated models. Moreover, the vertical axis intersection of Q2 must be zero or lower than zero [27]. The permutation test resulted in the highest R2 and Q2 values over other permutated models of R2 and Q2, and the intersection of Q2 was (0.0, −0.537). Therefore, the permutation test confirmed the validity of OPLS-DA model.

## 3. Materials and Methods 

### 3.1. Plant Materials

Rhizomes of *C. xanthorrhiza* were obtained from Sleman, Bantul, Kulon Progo (Yogyakarta), Karanganyar and Pati (Central Java), whereas rhizomes of *C. aeruginosa* were obtained from Yogyakarta. The authentication of rhizomes used was performed in Department of Pharmaceutical Biology, Faculty of Pharmacy, Universitas Gadjah Mada. These rhizomes were washed and then chopped into small and thin pieces. The chopped rhizomes were air dried at around 40–50 °C eight hours a day for five days and then ground into fine powder. These powdered rhizomes were used as samples for TLC analysis and ^1^H-NMR measurements.

### 3.2. Thin Layer Chromatography Analysis

Samples and standard solutions of curcumin were applied to TLC plate of silica gel 60 F254 (Merck, Darmstadt, Germany) with the CAMAG Linomat 5 Sample Applicator using the following settings: the first application was, from the *x*-axis and *y*-axis, 10.0 and 10.0 mm, respectively; band length 3.0 mm; and track distance 9.0 mm. Chromatographic analysis was carried out in a flat bottom chamber up to elution distance of 100 mm, using chloroform:methanol:formic acid (94:3:3 *v*/*v*) as the mobile phase. After the elution finished, the plate was scanned using CAMAG TLC Scanner 4 (CAMAG, Muttenz, Switzerland) with slit dimensions of 5.00 × 0.20 mm and scanning speed 20 mm/s. Curcumin contents were measured in absorption mode at UV wavelength 427 nm. Linomat 5 Sample Applicator and TLC Scanner 4 were controlled by winCATS Planar Chromatography Manager software SN 2110W018 V1.4.9 (CAMAG, Muttenz, Switzerland).

### 3.3. Analytical Method Validation

The TLC method was validated for selectivity, linearity and range, detection limit and quantitation limit, accuracy, and precision according to the International Conference on Harmonization (ICH) Q2(R1). The detection limit and quantitation limit were based on the standard deviation of the response and the slope [28]. For preparation of the standard solution used during validation, 10 mg of curcumin was accurately weighed and transferred to a 10.0 mL volumetric flask, dissolved, and diluted to volume with methanol to obtain the standard solution of curcumin with the concentration of 1000 mg/L. An intermediate standard solution with the curcumin concentration of 50 mg/L was prepared by transferring 0.25 mL of a curcumin standard solution into a 5.0 mL volumetric flask and diluting it to volume with methanol. For linearity evaluation, five different concentrations of calibration standard of curcumin were obtained by spotting each of 5, 6, 7, 8, and 9 µL of the intermediate standard solution on a TLC plate. The amounts of curcumin in the calibration standard series obtained were 250, 300, 350, 400, and 450 ng respectively. 

### 3.4. Sample Preparation for TLC Analysis

A 100 mg amount of *C. xanthorrhiza* rhizomes from each region was accurately weighed and placed into a 2 mL microtube, and then 1.5 mL methanol was added. The mixtures were vortexed for 5 min and then followed by centrifugation at 4000 rpm for 5 min. To prepare sample stock solution, 1 mL supernatant from the previous extraction was transferred to a 10.0 mL volumetric flask and diluted to volume with methanol. Test solutions were prepared by transferring 2.0 mL of each sample stock solution of *C. xanthorrhiza* extract from Bantul, Kulon Progo, and Karanganyar, and transferring 4.0 mL of each sample stock solution of *C. xanthorrhiza* extract from Sleman and Pati, into respective 5.0 mL volumetric flasks and diluting to volume with methanol. Binary mixtures of *C. xanthorrhiza* from Sleman with various concentrations (10%, 25%, 40%, 50%, and 75%) of *C. aeruginosa* as adulterant were also prepared. Extraction of each binary mixture was done according to the previous step. Sample stock solution for binary mixture was prepared by transferring each 1.0 mL supernatant from the previous extraction to 10.0 mL volumetric flask for mixtures of 10% and 25% *C. aeruginosa*, and transferring each 1.0 mL supernatant from the previous extraction to respective 5.0 mL volumetric flasks for mixtures of 40%, 50%, and 75% *C. aeruginosa*, and then diluting to volume with methanol. 

### 3.5. Sample Preparation for ^1^H-NMR Measurements

Pure dried powder samples of *C. xanthorrhiza* from each region, *C. aeruginosa*, and binary mixtures of *C. xanthorrhiza* with various concentrations of *C. aeruginosa* were prepared for ^1^H-NMR measurements. Binary mixtures were prepared by mixing *C. xanthorrhiza* from Sleman with various concentrations (10%, 25%, 40%, 50%, and 75%) of *C. aeruginosa* as adulterants in a total weight of 5 g.

### 3.6. ^1^H-NMR Measurements

The method used was according to Kim et al. [15] with slight modifications. In total, 25 mg of each dried powder samples (pure and binary mixture) was placed in a separate 2 mL microtube and then supplemented with 0.5 mL of CD_3_OD and 0.5 mL of KH_2_PO_4_ buffer (pH 6.0) in D_2_O containing TSP 0.01%. The mixture was vortexed (Vortex Mixer Maxi Mix^TM^ II, Thermo Fisher Scientific, Waltham, MA, USA) for 1 min followed by ultrasonication for 20 min and centrifugation (Centrifuge MPW-260, MPW Med. Instruments, Warsaw, Poland) at 13,000 rpm for 10 min to obtain a clear supernatant. Approximately of 0.8 mL of each supernatant was transferred to an NMR tube and immediately subjected to ^1^H-NMR measurements using a preset setting for all the samples. The ^1^H-NMR measurements were implemented using a 500 MHz NMR Spectrometer (JEOL ECZR, JEOL Ltd., Tokyo, Japan).

### 3.7. Bucketing of ^1^H-NMR Spectra and Multivariate Analysis

^1^H-NMR data processing was according to Gad and Bouzabata [29] with modifications. The ^1^H-NMR spectra were manually phase corrected, and subsequently, baseline correction was performed. Baseline correction was performed using polynomial fit in third order mode. The spectra were then normalized using total area before being binned to obtain ASCII data. The normalized ^1^H-NMR spectra were automatically binned to ASCII files using MestreNova software (version 12.0, Mestrelab Research, Santiago de Compostela, Spain). Spectra intensities were reduced to buckets with the spectral width (δ 0.04) forming a region of 0.08–10.04 mg/L and the total variables of 250 chemical shifts bin were generated for each ^1^H-NMR spectrum. The generated ASCII files were subjected to multivariate analysis. Before performing PCA and OPLS-DA, the data were scaled using Pareto scaling. PCA and OPLS-DA were performed using SIMCA 14.0 software (Sartorius, Malmo, Sweden). OPLS-DA was validated using permutation test to confirm the validity of OPLS-DA model.

## 4. Conclusions

TLC analysis and ^1^H-NMR-based metabolite fingerprinting coupled with multivariate analysis of PCA and OPLS-DA were successfully used for authentication and discrimination of *C. xanthorrhiza* extract. TLC analysis allows determination of curcumin contents of *C. xanthorrhiza* extract which could also be used to assess indication of adulteration of this extract. ^1^H-NMR-based metabolite fingerprinting coupled with PCA and OPLS-DA facilitated consistent discrimination among pure *C. xanthorrhiza* extract from adulterated ones.

## Figures and Tables

**Table 1:** Results of an accuracy and precision study using the standard addition method.

Addition Levels on the Sample	Analyte Taken (mg/L)	Analyte Found (mg/L)	Recovery (%)	SD	CV (%)
Low	9.99	10.19	101.97	0.59	5.77
Medium	12.49	12.49	100.00	0.39	3.15
High	14.99	15.16	101.16	0.80	5.26

**Table 2:** Results of curcumin analysis of the samples.

Sample		Analyte Found (%)	SD	CV (%)
*C. xanthorrhiza* from various regions	Karanganyar	1.15	0.01	0.00
	Sleman	1.02	0.01	0.99
	Bantul	1.06	0.01	0.95
	Kulon Progo	1.23	0.01	0.82
	Pati	0.74	0.01	1.36
*C. xanthorrhiza* adulterated with various concentrations of *C. aeruginosa*	0%	1.02	0.01	0.99
	10%	0.96	0.03	3.14
	25%	0.86	0.01	1.17
	40%	0.69	0.02	2.90
	50%	0.43	0.01	2.35
	75%	0.27	0.00	0.00
